# Nutrient Composition Analysis of Maize Hybrids Affected by Different Nitrogen Fertilisation Systems

**DOI:** 10.3390/plants11121593

**Published:** 2022-06-16

**Authors:** Csaba Bojtor, Seyed Mohammad Nasir Mousavi, Árpád Illés, Farid Golzardi, Adrienn Széles, Atala Szabó, János Nagy, Csaba L. Marton

**Affiliations:** 1 Institute of Land Use, Engineering and Precision Farming Technology, Faculty of Agricultural and Food Sciences and Environmental Management, University of Debrecen, 138 Böszörményi St., H-4032 Debrecen, Hungary; nasir@agr.unideb.hu (S.M.N.M.); illes.arpad@agr.unideb.hu (Á.I.); szelesa@agr.unideb.hu (A.S.); szabo.atala@agr.unideb.hu (A.S.); nagyjanos@agr.unideb.hu (J.N.); marton.csaba@atk.hu (C.L.M.); 2 Seed and Plant Improvement Institute, Agricultural Research, Education and Extension Organization (AREEO), Karaj P.O. Box 31585-4119, Iran; f.golzardi@areeo.ac.ir; 3 Eötvös Loránd Research Network, Centre for Agricultural Research, Agricultural Institute, 2 Brunszvik St., H-2462 Martonvásár, Hungary

**Keywords:** nutrient, NPK fertilizer, maize, principal component

## Abstract

Maize is one of the most widely used plants in the agricultural industry, and the fields of application of this plant are broad. The experiment was conducted at the Látókép Crop Production Experimental Station of the University of Debrecen, Hungary. Three mid-ripening maize hybrids with different FAO numbers were used in the present study. The effects of different nitrogen supplies were examined as a variable rate of abiotic stress and the interrelationship among the essential nutrients through the nutrient acquisition and partitioning of the different vegetative and generative plant parts. The results showed that NPK application compared to the control treatment (no fertilizer application) increased DM in all tissues of maize, while increasing nitrogen application from 120 to 300 kg ha^−1^ had no significant effect on this trait. The highest protein content was obtained with the nitrogen application of 120 kg ha^−1^, and the higher nitrogen fertilizer application had no significant effect on this trait. Seeds and leaves had a maximum zinc and manganese value in terms of nitrogen content (protein). Dry matter was positively correlated with nitrogen, potassium, and manganese content, while the dry matter had a negative correlation with nickel content. In general, to achieve a maximum quantitative and qualitative yield, it is recommended to use NPK fertilizer with a rate of 120 kg ha^−1^ N for maize cultivation.

## 1. Introduction

Maize is the third most important agricultural product globally after wheat and barley. Also, the amount of maize produced each year is more than other grains [1]. Maize is a monocotyledonous plant, and its edible part is the pomegranate that produces the seed of the same fruit [2]. Maize is considered an important food source in many different parts of the world. In addition, animal feed, biofuels, and raw materials in the industry are other widespread uses of maize [3]. Elemental deficiency is seen in a wide area of agricultural lands worldwide, resulting in reduced performance and reduced product quality. Elemental deficiency negatively affects the plant in terms of vascular system, root development, confectionery transport, carbohydrate combustion, nucleic acid synthesis, and pollen grain growth [4]. On the other hand, too much concentration in the plant causes poisoning and reduces the fertility and inoculation of maize. Too much or too little N and P in the maize plant interferes with the absorption of the other elements, such as zinc, iron, copper, and magnesium. A lack of elements causes a deformed state and infills the formed grains [5]. High yields in maize are not possible without chemical fertilizers (N, P, K) and livestock. By increasing the yield per hectare, the amount of absorption of these elements per ton of grain to the plant organs decreases. Most of the nutrients are absorbed until the beginning of seed formation [6,7,8]. Micronutrient fertilizers make up 4% of the total fertilizers used globally. The three micronutrients—iron, zinc, and manganese—are more involved in the nutrition of maize than other elements. Iron is one of the most important elements in redox reactions in plants. About 75% of the iron in the cell is associated with chloroplasts, a deficiency of which can stop the production of chloroplasts [9]. Chlorophyll production and maintenance, cellular respiration, chemical reduction of nitrate and sulfate, and nitrogen uptake are effective in nucleic acid, chloroplast, and RNA metabolism. The total amount of iron is 200 to 100,000 mg/kg in soils, but only a small amount is soluble. Unlike many other trace elements, zinc has a well-known role in plant systems [10]. Observations show that zinc deficiency shortens the distance between internodes. Metal cations including copper, iron, and manganese inhibit the uptake of zinc by plants. This phenomenon may occur due to competition for the occupation of the same carrier places [11]. On the other hand, high soil phosphorus can inhibit zinc uptake, especially in calcareous soils. Still, nitrogen fertilizers increase the need for zinc because it increases plant growth. Iron deficiency can generally be affected by high levels of other trace elements [12]. Increasing quantitative and qualitative performance requires essential consumption elements [13]. Micronutrients are essential for plant growth but are needed in much smaller amounts than essential nutrients such as nitrogen, phosphorus, sulphur, and potassium [14,15]. Micronutrients such as zinc and copper are essential for plants, humans, and animals. Increasing the concentration of micronutrients in major crops greatly improves human nutrition globally [16,17]. Iron is one of the most important components of the plant enzyme system. It is also one of the components of the protein ferredoxin, which is necessary for reducing nitrate and sulfate and the assimilation of nitrogen and energy production. Iron is a catalyst or part of the enzymatic system that produces chlorophyll. In the synthesis of chlorophyll, iron plays a role as a coenzyme in three basic steps. The balance of iron distribution is disturbed in the case of iron deficiency in plants in chloroplasts. Therefore, a deficiency of this element reduces chlorophyll synthesis and eventually leaves yellowing [18]. Today, studies show that iron is involved in protein synthesis and root tip growth. Sulphur plays an important role in producing proteins required for plant growth. Although zinc is required in small amounts by the maize plant and is a micronutrient, zinc plays an important role in forming plant enzymes. Zinc also plays an essential role in plant metabolism [19,20,21]. A state-of-the-art fertilization advisory system cannot be done without a diagnostic plant analysis in addition to soil testing [22]. Although plant analysis during the growing season is not suitable for determining the fertilizer dose, it can be used in many areas of plant nutrition: (1) to determine the nutritional status of the plant; (2) for soil testing, nutrient supply, and fertilization practices; (3) to detect nutrient interactions; (4) to detect latent eating disorders and to identify a visible deficiency or overweight symptoms; (5) to explore the causes of developmental disorders; and (6) to predict the yield and quality [23,24]. The concentration of nutrients is important. Maintaining balance in plant nutrients is essential because nutrients such as nitrogen and phosphorus play an important role in maintaining water efficiency. An element such as potassium controls water loss from the plant [25]. The main aim was to evaluate the complex nutrient composition of maize to determine the best fertilizer management strategy to enhance the nutrient balance of the main plant parts (stem, cob, leaf, and grain) in this research.

## 2. Results

A variance analysis showed that the NPK fertilizer effect was significant on dry matter, nitrogen, phosphorus, potassium, magnesium, sulphur, zinc, iron, copper, and manganese. Also, the genotypes effect was significant on dry matter, nitrogen, phosphorus, potassium, magnesium, calcium, iron, copper, manganese, and molybdenum. The tissue effect had a significant effect on dry matter, nitrogen, phosphorus, potassium, magnesium, calcium, nickel, sulphur, iron, copper, manganese, and molybdenum in this research. The NPK in genotype interaction effect was significant on dry matter, calcium, zinc, and manganese. The NPK in tissue interaction effect was significant on dry matter, potassium, magnesium, calcium, sulphur, zinc, copper, and manganese. The genotype in tissue interaction effect was significant on dry matter, phosphorus, magnesium, calcium, nickel, sulphur, and zinc. The NPK in genotypes in tissue interaction effect was significant on calcium and zinc (Table 1). In grouping for the Tukey analysis, the results showed that dry matter, nitrogen, phosphorus, zinc, and molybdenum resulted in a maximum amount of grain tissue. Potassium had a maximal effect on stem tissue. Sulphur, copper, magnesium, manganese, calcium, and iron had a maximal effect on leaf tissue. Nickel had a maximal effect on cob maize. This study showed that dry matter, potassium, nitrogen, magnesium, zinc, phosphorus, calcium, and iron had a minimal effect on cob maize. There was a maximal effect on the grain and leaves of maize due to nitrogen because nitrogen helps to store dry matter in the grain and helps the leaves to photosynthesize. There was a maximal effect on the grain of maize—as well as the leaves, stem, and cob—due to phosphorous. There was a maximum effect on the stem tissue of maize due to potassium because potassium strengthens the stem against strong winds—it also had a maximal effect on the leaves; grain and cob have similar amounts of potassium. There was a maximal effect on the leaves of maize due to magnesium, as well as a maximal effect on the stem, grain, and cob. Calcium had a maximal effect on the leaves, and, after that, on the stem, grain, and cob of maize. Similarly, sulphur had a maximum effect on the leaves, and, after that, on the grain, stem and cob. There was a maximal effect on the grain due to zinc, but in the leaf, stem, and cob there was a similar effect. There was a maximal effect on the leaves of the plant, as well as the stem. Iron had a similar effect on the grain and cob of the plant. Copper had a maximal effect on the leaf and the stem of the plant. Also, copper had a similar effect on cob maize and grain. There was a maximal effect on the leaves and grain of maize due to manganese, as well as the cob maize and stem. Molybdenum had a maximal effect on the grain and had similar effects on cob and leaves of the plant. Nickel had a maximal effect on the cob of the plant. Nickel had similar effect on the stem, grain, and leaves (Table 2).

**Figure 1:**
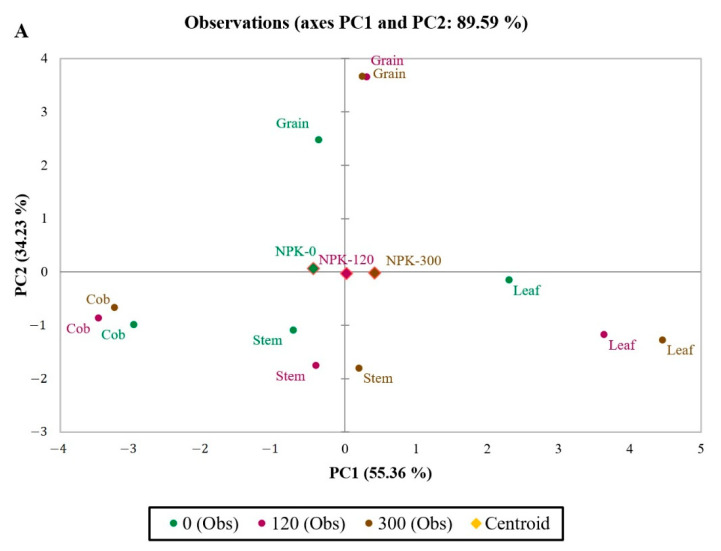

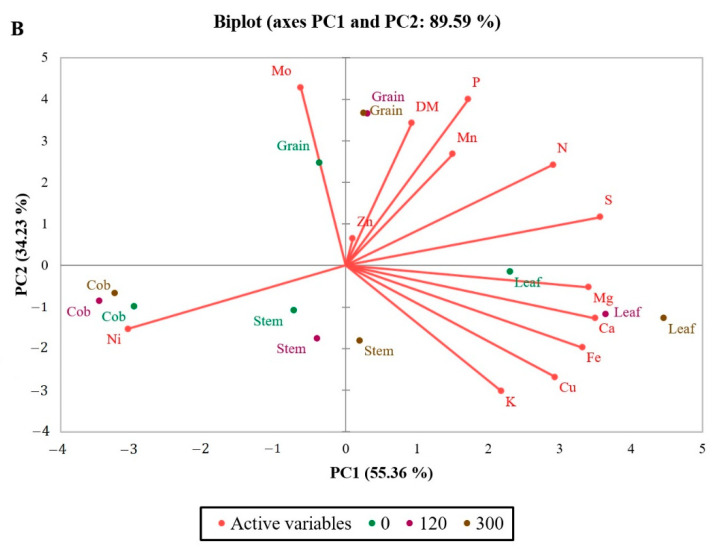
Principal component analysis (PCA) observation plot (**A**) and biplot (**B**) of the first two components performed on dry matter production and nutrient composition of maize tissues affected by fertilizer levels. Centroids showing segregation of maize tissues and fertilizer levels on axis PC1 and PC2 based on dry matter production and nutrient composition. (N (nitrogen), P (phosphorus), DM (dry matter), K (potassium), Mn (manganese), S (sulphur), Mo (molybdenum), Mg (magnesium), Ca (calcium), Fe (iron), Cu (copper), Ni (nickel)).

The principal component analysis resulted in the identification of traits (dry matter production and nutrient composition), which are presented in Figure 1, of different plant tissues of maize under the influence of fertilizer levels. The results of this PCA based on the correlation matrix indicated that the two main principal components with eigenvalues more than 1 (eigenvalues > 1) were related to about 90% of the total variation in the traits. The first principal component (PC1) had a significant positive effect on magnesium, calcium, sulphur, nitrogen, iron, and copper. Also, PC1 had a significant negative effect on nickel. The second principal component (PC2) had a significant positive effect on dry matter, phosphorus, Mo, and manganese, and a significant negative effect on potassium.

Based on this principal component analysis, increasing the fertilizer application was not significant for the first principal component (Figure 1A). The biplot results (Figure 1B) on the effect of fertilizer levels on leaf traits indicated that, with increasing fertilizer application, the values of the first principal component have increased (variation in the second principal component did not have a significant on the effect of the fertilizer on leaf traits). Therefore, fertilizer levels of 300 and 120 kg ha^−1^ increased the content of magnesium, calcium, sulphur, nitrogen, iron, and copper in the leaves compared to the control treatment (without fertilizer application). The biplot results (Figure 1B) for the effect of fertilizer levels on grain traits indicated that fertilizer application had increased the values of the second principal component (variation in the first principal component was not significant for the effect of the fertilizer on grain traits). Therefore, fertilizer levels of 300 and 120 kg ha^−1^ have increased the amount of dry matter, phosphorus, molybdenum, and manganese content in the grain compared to the control treatment (without fertilizer application). In addition, increasing the fertilizer application increased the amount of the first principal component in the case of the stem, but this increase was not significant. Also, the application of different fertilizer levels had no significant effect on cob traits (Figure 1B).

**Figure 2:**
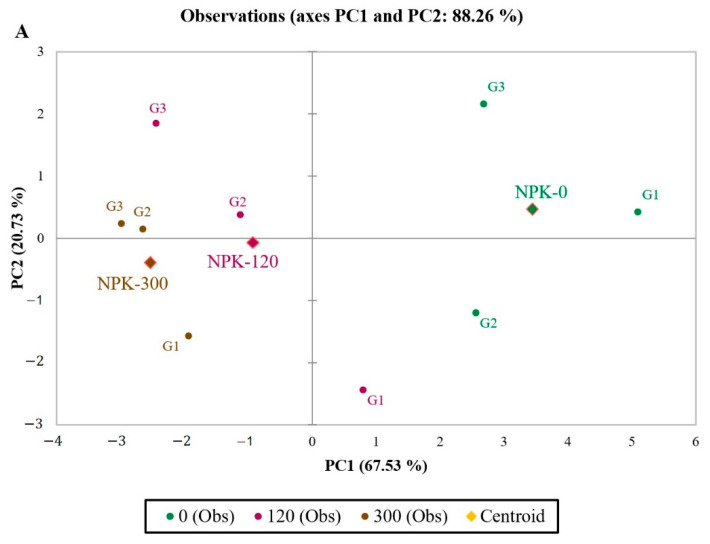

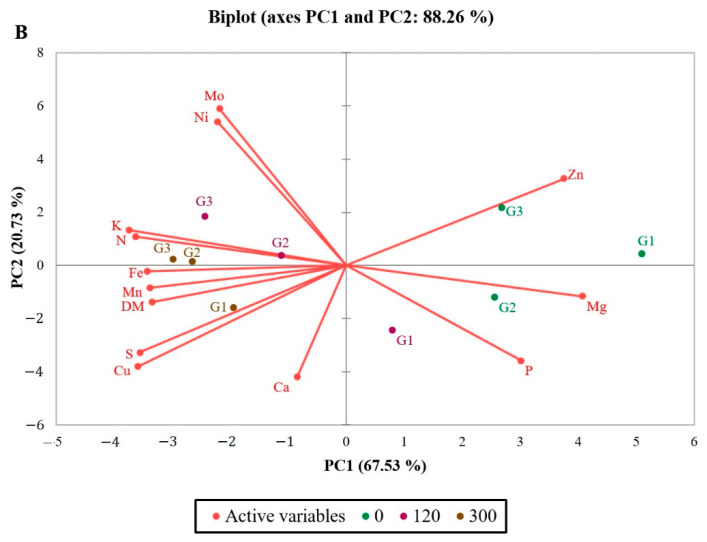
Principal component analysis (PCA) observation plot (**A**) and biplot (**B**) of the first two components performed on dry matter production and nutrient composition of maize genotypes affected by fertilizer levels. Centroids showing segregation of maize genotypes. (G1: FAO 420; G2: FAO 490; G3: FAO 390). (N (nitrogen), P (phosphorus), DM (dry matter), K (potassium), Mn (manganese), S (sulphur), Mo (molybdenum), Mg (magnesium), Ca (calcium), Fe (iron), Cu (copper), Ni (nickel)).

The analysis of the principal component results is presented in Figure 2 of the studied traits (dry matter production and nutrient composition) in different maize genotypes under fertilizer levels. This principal component analysis based on a correlation matrix indicated that the two principal component’s analysis with eigenvalues more than 1 (eigenvalues > 1) were related to about 88% of the total variation in the studied traits. The first principal component (PC1) had a significant positive effect on magnesium, zinc, and phosphorus, and a significant negative effect on iron, manganese, dry matter, nitrogen, potassium, sulphur, and copper. The second principal component (PC2) had a significant positive effect on molybdenum and nickel. Therefore, higher values of the first principal component indicate higher magnesium, zinc, and P elements and a lower content of iron, manganese, dry matter, nitrogen, potassium, sulphur, and copper. In comparison, higher values of the second principal component indicate a higher content of molybdenum and nickel. Based on this principal component analysis results, increasing fertilizer application decreased the first and second principal components (Figure 2A). A biplot of the effect of fertilizer levels on the studied genotypes indicated that increasing fertilizer use in all three genotypes causes decreased values of the first principal component. Therefore, fertilizer levels of 300 and 120 kg ha^−1^ caused an increase in dry matter and concentrations of iron, manganese, nitrogen, potassium, sulphur, and copper in all hybrids compared to the control treatment (without fertilizer application). The highest amount of the first principal component was observed in all three fertilizer levels in the FAO 420 genotype. This hybrid has the lowest dry matter and concentration of iron, manganese, nitrogen, potassium, sulphur, and copper compared to the other two hybrids (Figure 2B).

## 3. Discussion

Variance analysis showed variation in the NPK fertilizer and Genotype effect on dry matter, nitrogen, phosphorus, potassium, magnesium, iron, copper, and manganese. Variations exist for tissue from all nutrients except zinc. Phelps et al. [26] showed that increasing nitrogen fertilizer causes an increased nitrogen concentration in some maize cultivars. Micronutrients are essential for plant growth but are needed in much smaller amounts than essential nutrients such as nitrogen, phosphorus, sulphur, and potassium [14]. The minimum difference between the genotypes was observed at the fertilizer level of 300 kg ha^−1^ in terms of quantitative and qualitative traits. The control treatment and the fertilizer level of 120 kg ha^−1^ showed more differences. In addition, it was observed that the use of NPK fertilizer (N1) compared to the control treatment (N0) had a significant effect on the studied traits. In contrast, applying nitrogen fertilizer at a rate of 300 kg ha^−1^ (N2) compared to treatment N1 does not make much difference. Applying nitrogen fertilizer of more than 120 kg ha^−1^ had little effect on the studied traits. Grain had the maximum value for phosphorus and zinc; stem tissue had a maximum value for potassium; the leaves had the maximum value for magnesium, iron, manganese, and calcium; and cob had the maximum nickel value. Santos et al. showed that the response of the maize yield to micronutrients is due to a specific place or season [27]. Micronutrient nutrition cases increase the forage and grain yield in maize, among which the positive role of iron and zinc increase the yield more than manganese. Thus, research shows that plant height and yield and stem increased under the zinc fertilizer and improved the amount of dry matter and yield. Several factors are involved in the development of iron deficiency [28,29,30]. Bicarbonate is one of the main factors in iron deficiency, so some researchers consider its role more important than other factors. Also, research showed that the zinc fertilizer, either through leaves or through the soil, had a significant effect on increasing the dry weight of maize compared to control treatments. On the other hand, the application of zinc through the soil can be effective for future crops [31,32,33]. The results of this PCA based on the correlation matrix revealed that the first two principal components with eigenvalues > 1 accounted for 89.59% of the total variation in traits. Therefore, higher values of the first principal component indicate a higher content of magnesium, calcium, sulphur, nitrogen, iron, and copper elements. Increasing fertilizer levels had increased the amount of dry matter and concentrations of iron, manganese, nitrogen, potassium, sulphur, and copper in the biomass of maize genotypes. The dry matter on FAO 490 and FAO 390 hybrids had maximum iron, manganese, nitrogen, potassium, sulphur, and copper concentrations in fertilizer of 300 kg ha^−1^. The results showed that the highest amount of fertilizer used in plant tissues was related to leaves. While cob and seeds showed the least variation against fertilizer treatments, the amount of dry matter showed a positive correlation with phosphorus, manganese, and nitrogen content, and a negative correlation with nickel. Nickel content was negatively correlated with other plant elements. The abundance of this element in maize plant tissues can cause a sharp decline in its quantitative and qualitative yield. Plant tissues, leaves, and seeds had the highest nutritional value, while cob with anti-nutritional elements was maize’s least valuable plant tissue. Maize is one of the crops that needs a lot of nitrogen. This element is often supplied as a chemical fertilizer. The plant properties of maize can be greatly affected by the amount of nitrogen available. As Sabir et al. [34] stated, the amount of nitrogen is one of the factors affecting the leaf surface development of each plant. The development of plant shading in maize and increasing the size and longevity of each leaf increases the leaf area index. This increase in the photosynthetic level of the plant can improve other growth indices and, ultimately, its yield. Nitrogen can affect other elements in maize growth stages. As a result of genetic progress, breeding, and the permanent development of agrotechnics, the potential productivity and yield of maize is on the rise. Thus, it is especially important to check, clarify, and determine the nutrient concentration limits for high yields. Long-term experiments offer a good opportunity for this, where the soil nutrient supply varies over a wide range [35]. Increasing the amount of nitrogen affects the absorption of other elements such as potassium, magnesium, calcium, and phosphorus, and, in some cases, intensifies the absorption of some elements [36]. The final grain yield consists of two physiological processes: photosynthesis and second remobilization of the accumulated material before flowering. In other words, in post-pollination cereals, seeds are very active destinations for carbon, micro-, and macronutrient uptake [37]. Macro- and micronutrients are needed for growth in plants, cause freshness and keep the plants green, and cause the rapid growth of foliage. It also affects the quantity and quality of the product, such as the colour, grain and fruit size, sugar content, and essential amino acids. The amount of macronutrients and micronutrients absorbed in plants indicates the status of macro- and micronutrients during the growth period of the plant. Increasing the efficiency of these elements increases the absorption of these elements in dry matter production and yield [38,39].

## 4. Materials and Methods

### 4.1. The Field Experiment

**Figure 3:**
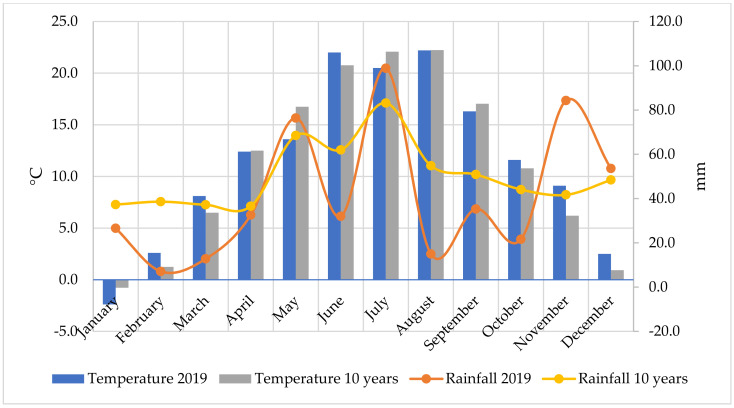
Temperature and precipitation data of 2019 compared to the 10-year average.

The experiment was conducted at the Látókép Crop Production Experimental Station of the University of Debrecen, Hungary (47°33′ N, 21°26′ E, 111 m asl) in 2019, with relatively hot weather and fluctuating precipitation compared to the 10-year average values (Figure 3).

Three mid-ripening maize hybrids (*Zea mays* L. G1: FAO 420; G2: FAO 490; G3: FAO 390) with different FAO numbers were used in the present study. The effects of different nitrogen supplies as a variable rate of abiotic stress and the interrelationship among the essential nutrients were examined through the nutrient acquisition and partitioning of the different vegetative and generative plant parts. The soil type of the experimental field area is calcareous chernozem, with a humus content (Hu%) of 2.7–2.8 value; pH (KCl): 6.46; Arany-type plasticity index: 43.3; total nitrogen content: 6.2 mg kg^−1^; magnesium: 332.4 mg kg^−1^; sulphur: 2.81 mg kg^−1^; potassium: 199.06 mg kg^−1^; sodium: 38 mg kg^−1^; phosphorus: 58.21 mg kg^−1^; copper: 5.86 mg kg^−1^; manganese: 438 mg kg^−1^; and zinc: 2.8 mg kg^−1^ [39]. The examined plots were part of a 38-year, long-term multifactorial nitrogen fertilisation experiment in a maize monoculture field, which allowed for studying and evaluating the long-lasting effects of different nitrogen fertilisation systems (Table 3). The fertilizer reactions of the different maize genotypes can be examined in a stable field environment, with documented agronomic history that is identical in every year for 38 years. Phosphorus and potassium doses were applied during autumn base fertilization, using potassium-chloride and superphosphate, respectively. Nitrogen fertilization was applied before sowing on 05/04/2019, without splitting using calcium-ammonium nitrate fertilizer. The soil parameters of the experimental area are suitable for the prevention of nitrogen losses.

The autumn base fertilisation of phosphorus and potassium was applied on 31/09/2018, then winter ploughing was used for soil tillage on 05/10/2018. The sowing date was 20/04/2019, using a 7.6 m^2^ experimental plot size with a density of 73,000 plant ha^−1^. Herbicide treatments were applied on 02/05 and 23/05, row cultivation on 27/05, and the harvest was on 16/10/2019. During the vegetation period, five main phenological phases (V2, V4, V8, VT, and R6 stage, respectively) were used for nutrient composition analyses. Four average, representative plants were collected at each sampling time in each of the three NPK levels. All plant samples were separated into leaves and stalks—and at the R6 stage—cob and grain, then dried at 60 °C to a constant weight, which was weighed to obtain the dry matter of the plants (DM) and ground into fine powder. The complex nutrient composition of the different plant parts was determined in an accredited laboratory using a microwave-assisted multi-element analysis [40]. For the complex nutrient composition, the concentrations of essential nutrients were detected. The amount of phosphorus (P), potassium (K), magnesium (Mg), calcium (Ca), sulphur (S), iron (Fe), manganese (Mn), zinc (Zn), copper (Cu), molybdenum (Mo), and nickel (N) were quantified using ICP-OES and ICP-MS, respectively.

### 4.2. Sample Analysis

The sample preparation for the laboratory analysis was as follows: two parallel measurements were made from each sample. A 0.4 g sample was weighed, and then 2 mL of high purity water and 4 mL of cc. nitric acid were added to the samples. According to the Milestone Ultrawave microwave system manual (Milestone Srl, Sorisole, Italy), sample extraction with microwave digestion was performed at 200 °C with a holding time of 10 min. ICP-OES analysis was used to determine the phosphorus, potassium, magnesium, calcium, sulphur, iron, and manganese concentration. A sample of 5 mL was pipetted into a plastic test tube, and 5 mL of deionized water, 0.2 mL of the acid mixture, and 0.2 mL of 100 ppm Y-containing ISTD (internal standard) were added to the sample. The mixture was then homogenized and put into the 5900 ICP-OES (Agilent Technologies Inc., Santa Clara, CA, USA). The samples’ zinc, copper, molybdenum, and nickel concentrations were determined from the homogenized mixture with 7900 ICP-MS (Agilent Technologies Inc., Santa Clara, CA, USA). Extracted samples of 5 mL, 1 mL of the acid mixture, and 4 mL of deionized water were added to the test tube for the analysis. A sample of each matrix type was prepared twice, and blank samples were prepared in each series of measurements by measuring water of the same quantity instead of a sample.

The nitrogen concentration was measured using the Dumas combustion method [41]. The samples were subjected to oxidative digestion at a high temperature (900 °C) with a controlled oxygen supply. The resulting flue gases passed through a copper oxide-platinum catalyst using a CO_2_ carrier gas, thus ensuring complete oxidation. After the reduction processes and the carrier gas purification, the nitrogen content remaining in the CO_2_ carrier gas was detected in a thermal conductivity detector (VELP NDA 702, Velp Scientifica Srl, Usmate, Italy). The N_2_ volume provided an electrical measurement signal, from which the nitrogen content of the various burned samples was measured and calculated based on a pre-prepared calibration curve.

### 4.3. Statistical Analysis

One of the most practical statistical methods in data analysis is ANOVA’s “variance analysis” technique. In this method, the total variance of the data is divided into two or more parts based on one or more factor variables. Based on tests related to variance, groups can be tested for homogeneity or heterogeneity. Tukey’s least real difference tests is also famously used as a studentized statistic for all pairwise comparisons between groups and matches the experimental error rate with the collection error rate for all pairwise comparisons. The principal component analysis (PCA) technique is a statistical method often used to examine a group of correlated variables. A multivariate statistical analysis method selects a smaller number of factors called principal components from among the primary factors so that the insignificant data are removed. The first basic component extracted considers the largest amount of data scattered in the entire dataset. This means that the first component is correlated with a number of variables. The second extracted component has two important features. First, this component considers the largest dataset that the first component had not computed, and second, it is not correlated with the first component. In other words, regardless of the previous component, each component describes a smaller variance by passing from the initial component to the terminal components. The first principal component always describes the maximum variance, and the last component describes the least variance. A lot of information will not be lost by deleting the last component. Minitab and Genstat software were used for the analysis in this study.

## 5. Conclusions

NPK application compared to control treatment (no fertilizer application) increased DM in all tissues of maize, while increasing the nitrogen application from 120 to 300 kg ha^−1^ had no significant effect on this trait. However, the nitrogen content of maize stalks and leaves increased linearly with increasing nitrogen fertilizer application. The application of nitrogen fertilizer of more than 120 kg ha^−1^ does not seem to have a small effect on yield, and by being stored in leaves (mainly) and stems (in part), it improves the crude protein content and improves the quality of maize forage. The highest protein content of grain was obtained with the application of 120 kg ha^−1^ N and a higher application of nitrogen fertilizer had no significant effect on this trait. In addition, increasing fertilizer consumption increases the content of potassium (stems and leaves), calcium (stems), sulphur (all tissues), iron (leaves and seeds), copper (leaves, stems, and cobs), and manganese (leaves, seeds, and cob), while it decreased in response to an increased fertilizer application for the content of magnesium (leaves, stems, and cobs), zinc (all tissues), molybdenum (leaves), and nickel (grain). The highest content of phosphorus and molybdenum was found in seeds with the highest phosphorus and molybdenum, stems had the highest content of potassium, and cob had the highest nickel content. However, the maximum content of magnesium, calcium, sulphur, iron, and copper was present in maize leaves. Seeds and leaves had zinc and manganese in their nitrogen content (protein). Among plant tissues, leaves showed the highest reaction to the amount of fertilizer used and the content of all its elements, except calcium and nickel, were affected by fertilizer treatment. In contrast, cob and seed showed the least variation and fluctuations. It seems that maize with intrinsic mechanisms prevents severe changes in grain composition as much as possible to increase the stability of the next generation and the possibility of its survival. The amount of dry matter had a positive correlation with the nitrogen, potassium, and manganese content, and a negative correlation with the nickel content in maize. Nickel had a significant negative correlation with all elements in the plant (except molybdenum). It seems that the high heavy metal content can reduce the quantitative and qualitative yield of maize. Therefore, it is better to avoid maize cultivation in nickel-contaminated soils. In general, to achieve a maximum quantitative and qualitative yield of maize grain while considering environmental issues, it is recommended to use NPK fertilizer with a rate of 120 kg ha^−1^ N for the cultivation of this plant.

## Figures and Tables

**Table 1:** Variance analysis of plant parts (tissues) of different maize hybrids for NPK fertilizer levels.

		Df	F			Df	F
DM	NPK	2	90.25 **	S	NPK	2	51.89 **
	Genotype	2	11.8 **		Genotype	2	1.45
	Tissue	3	4387.79 **		Tissue	3	397.53 **
	NPK * Genotype	4	4.91 **		NPK * Genotype	4	0.5
	NPK * Tissue	6	12.09 **		NPK * Tissue	6	11.77 **
	Genotype * Tissue	6	11.11 **		Genotype * Tissue	6	3.59 **
	NPK * Genotype * Tissue	12	1.48		NPK * Genotype * Tissue	12	1.8
N	NPK	2	6.45 **	Zn	NPK	2	83.46 **
	Genotype	2	2.38 *		Genotype	2	2.52
	Tissue	3	23.2 **		Tissue	3	1.74
	NPK * Genotype	4	0.85		NPK * Genotype	4	6.32 **
	NPK * Tissue	6	1.34		NPK * Tissue	6	5.41 **
	Genotype * Tissue	6	0.72		Genotype * Tissue	6	2.14 *
	NPK * Genotype * Tissue	12	0.71		NPK * Genotype * Tissue	12	2.22 *
P	NPK	2	3.31 *	Fe	NPK	2	10.96 **
	Genotype	2	5.8 **		Genotype	2	3.31 *
	Tissue	3	164.98 **		Tissue	3	185.68 **
	NPK * Genotype	4	1.89		NPK * Genotype	4	1.95
	NPK * Tissue	6	1.91		NPK * Tissue	6	2.13
	Genotype * Tissue	6	2.98 *		Genotype * Tissue	6	1.03
	NPK * Genotype * Tissue	12	1.23		NPK * Genotype * Tissue	12	1.67
K	NPK	2	14.36 **	Cu	NPK	2	66.4 **
	Genotype	2	3.87 *		Genotype	2	3.54 *
	Tissue	3	222.98 **		Tissue	3	250.45 **
	NPK * Genotype	4	1.05		NPK * Genotype	4	0.82
	NPK * Tissue	6	6.63 **		NPK * Tissue	6	17.67 **
	Genotype * Tissue	6	1.15		Genotype * Tissue	6	1.88
	NPK * Genotype * Tissue	12	1.02		NPK * Genotype * Tissue	12	0.6
Mg	NPK	2	14.99 **	Mn	NPK	2	7.1 **
	Genotype	2	7.25 **		Genotype	2	4.47 *
	Tissue	3	426.93 **		Tissue	3	107.91 **
	NPK * Genotype	4	1.24		NPK * Genotype	4	3.87 **
	NPK * Tissue	6	4.99 **		NPK * Tissue	6	6.6 **
	Genotype * Tissue	6	6.84 **		Genotype * Tissue	6	0.5
	NPK * Genotype * Tissue	12	0.84		NPK * Genotype * Tissue	12	0.86
Ca	NPK	2	0.43	Mo	NPK	2	0.85
	Genotype	2	10.53 **		Genotype	2	5.89 **
	Tissue	3	1442.54 **		Tissue	3	18.74 **
	NPK * Genotype	4	2.31 *		NPK * Genotype	4	1.14
	NPK * Tissue	6	2.58 *		NPK * Tissue	6	2.7
	Genotype * Tissue	6	6.26 **		Genotype * Tissue	6	2.08
	NPK * Genotype * Tissue	12	2.61 **		NPK * Genotype * Tissue	12	0.18
Ni	NPK	2	1.26				
	Genotype	2	3.07				
	Tissue	3	12.28 **				
	NPK * Genotype	4	1.3				
	NPK * Tissue	6	1.02				
	Genotype * Tissue	6	2.64 *				
	NPK * Genotype * Tissue	12	0.68				

** and * indicate significance at 1% and 5%.

**Table 2:** Grouping based on Tukey analysis of different parts of tissue.

	Tissue	Grouping		Tissue	Grouping		Tissue	Grouping
DM	Grain	A	N	Grain	A	P	Grain	A
	Stem	B		Leaf	A		Leaf	B
	Leaf	C		Stem	B		Stem	C
	Cob	D		Cob	C		Cob	D
K	Stem	A	Mg	Leaf	A	Ca	Leaf	A
	Leaf	B		Stem	B		Stem	B
	Grain	C		Grain	B		Grain	C
	Cob	C		Cob	C		Cob	D
S	Leaf	A	Zn	Grain	A	Fe	Leaf	A
	Grain	B		Leaf	B		Stem	B
	Stem	C		Stem	B		Grain	C
	Cob	D		Cob	B		Cob	C
Cu	Leaf	A	Mn	Leaf	A	Mo	Grain	A
	Stem	B		Grain	A		Cob	B
	Cob	C		Cob	B		Leaf	B
	Grain	C		Stem	C		Stem	C
Ni	Cob	A						
	Stem	B						
	Grain	B						
	Leaf	B						

**Table 3:** NPK fertilisation doses applied in the long-term multifactorial experiment.

Fertilisation Level	N (kg ha^−1^)	P (kg ha^−1^)	K (kg ha^−1^)
N0	0	0	0
N1	120	80.41	179.28
N2	300	80.41	179.28

## Data Availability

Not applicable.
